# Supplementary material for: Brillouin Klein bottle from artificial gauge fields
Source: Nat Commun. 2022 Apr 25;13:2215. doi: 10.1038/s41467-022-29953-7 (PMC9038716; doi:10.1038/s41467-022-29953-7)
Supplement: Supplementary file 1 — Supplementary Information [file 41467_2022_29953_MOESM1_ESM.pdf]

Supplemental Information for "Brillouin Klein Bottle From Artificial Gauge Fields"

Contents

Supplementary Note 1. Crystal Symmetries with Gauge Fields 1

Supplementary Note 2. The Flux Requirement for Momentum-space Glide Reflection Symmetry 1

Supplementary Note 3. Detailed derivation for the glide reflection operator 2

Supplementary Note 4. Screw Rotations in Momentum Space 3

Supplementary Note 5. Numerical Demonstration of Stability 6

Supplementary Note 6. Gauge Fields in Physical Systems 6

Supplementary Note 1. Crystal Symmetries with Gauge Fields

First of all, we present a formalism of crystal symmetries on a lattice with gauge fields. Only gauge flux configurations are gauge invariant. A given gauge flux configuration can be described by numerous corresponding gauge connection configurations. Reversely, two gauge connection configurations corresponding to the same flux configuration are related by a gauge transformation. A gauge transformation G simply multiplies a phase for each lattice site, and therefore can be regarded as a diagonal matrix indexed by the lattice sites.

The spatial symmetries are determined by the flux configuration, which is described by a chosen gauge connection configuration. A spatial symmetry R that preserves the flux configuration in general changes the connection configuration. The transformed connection configuration must be related to the original one by a gauge transformation G_R, since they correspond to the same flux configuration. Thus, the physical operator for the spatial symmetry R is given by

$$\mathsf{R} = \mathsf{G}_R R, \tag{1}$$

a combination of spatial symmetry R and the gauge transformation G_R.

We now consider how the physical operator R acts on a tight-binding model, $H = \sum_{ij} t_{ij} |i\rangle\langle j|$. The transformation of H is given by

$$\begin{aligned} H' &= \sum_{ij} t_{ij} |\mathsf{G}_R R(i)\rangle\langle \mathsf{G}_R R(j)| \qquad (2)\\ &= \sum_{ij} t_{ij} \mathsf{G}_R(R(i)) \mathsf{G}_R^*(R(j)) |R(i)\rangle\langle R(j)| \\ &= \sum_{ij} \mathsf{G}_R(i) \mathsf{G}_R^*(j) t_{R^{-1}(i)R^{-1}(j)} |i\rangle\langle j|, \qquad (3) \end{aligned}$$

where $G_R(i)$ is the phase for site i, and $R(i)$ is the site transformed from i by R. Thus, the invariance under R gives

$$t_{ij} = \mathsf{G}_R(i) \mathsf{G}_R^*(j) t_{R^{-1}(i)R^{-1}(j)}. \tag{4}$$

Supplementary Note 2. The Flux Requirement for Momentum-space Glide Reflection Symmetry

In this section, we show that the projective algebraic relation, Eq. (1) in the main text, leads to the requirement on the flux configuration as mentioned in the main text.

Supplementary Eq. (1) leads to

$$\mathsf{M}_x\mathsf{L}_y\mathsf{M}_x^{-1}\mathsf{L}_y^{-1} = \mathsf{G}_M M_x \mathsf{G}_y L_y (\mathsf{G}_M M_x)^{-1}(\mathsf{G}_y L_y)^{-1} \tag{5}$$

$$= \mathsf{G}_M (M_x \mathsf{G}_y M_x^{-1}) M_x L_y M_x^{-1} L_y^{-1} (L_y \mathsf{G}_M L_y^{-1}) \mathsf{G}_y^{-1}$$

$$= \mathsf{G}_M(\boldsymbol{r})\mathsf{G}_y(M_x(\boldsymbol{r}))\mathsf{G}_M^*(L_y^{-1}(\boldsymbol{r}))\mathsf{G}_y^*(\boldsymbol{r}). \tag{6}$$

Hence, the projective algebraic relation gives the identity,

$$\mathsf{G}_M(\boldsymbol{r})\mathsf{G}_y(M_x(\boldsymbol{r}))\mathsf{G}_M^*(L_y^{-1}(\boldsymbol{r}))\mathsf{G}_y^*(\boldsymbol{r}) = -1. \tag{7}$$

Consider a rectangle invariant under M_x and have a unit lattice length along the y-direction as Supplementary Fig.1a. Symmetry M_x and L_y constrain the hopping amplitudes by

$$t_{24} = \mathsf{G}_M(2)\mathsf{G}_M^*(4)t_{13}, \quad t_{34} = \mathsf{G}_y(3)\mathsf{G}_y^*(4)t_{12}. \tag{8}$$

Then, the flux through the rectangle satisfies

$$e^{-i\Phi} = e^{i\phi_{12}}e^{i\phi_{24}}e^{i\phi_{43}}e^{i\phi_{31}} \tag{9}$$

$$= \mathsf{G}_M(2)\mathsf{G}_M^*(4)\mathsf{G}_y^*(3)\mathsf{G}_y(4)e^{i\phi_{12}}e^{i\phi_{13}}e^{i\phi_{21}}e^{i\phi_{31}}$$

$$= \mathsf{G}_M(2)\mathsf{G}_M^*(4)\mathsf{G}_y^*(3)\mathsf{G}_y(4)$$

$$= \mathsf{G}_M(L_y^{-1}(4))\mathsf{G}_M^*(4)\mathsf{G}_y^*(M_x(4))\mathsf{G}_y(4) = -1. \tag{10}$$

Thus, we have proved the flux requirement, i.e., the magnetic flux through this rectangle is π.

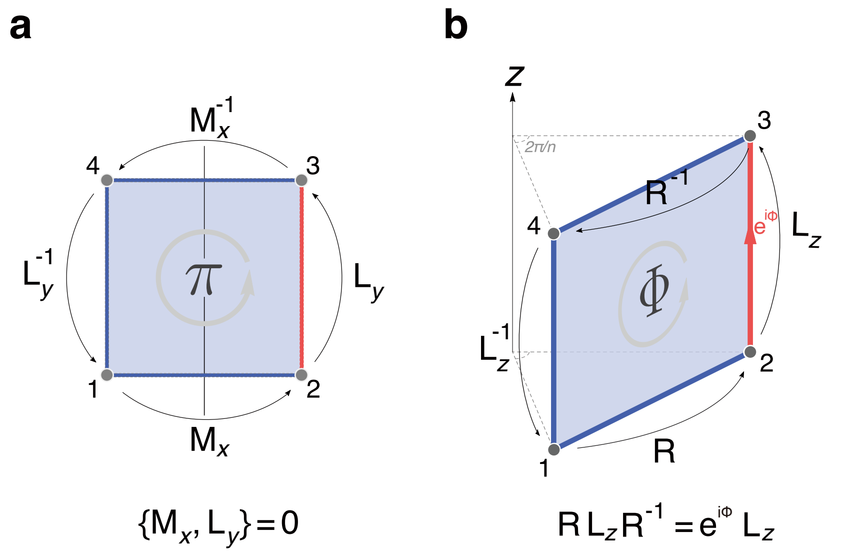

Supplementary Fig.1. **Flux configurations for the projective algebraic relations**. **a** The flux through a rectangle with unit lattice length along the y direction and invariant under the reflection. We choose the gauge condition: All blue bonds have a hopping phases $+1$, and red bond has a hopping phase -1. **b** Flux through a rectangle spanned by translation and the rotation. We choose the gauge condition: All blue bonds have a hopping phase 1, and the red bond has a hopping phase $e^{i\Phi}$.

Supplementary Note 3. Detailed derivation for the glide reflection operator

We now derive the glide reflection operator of the model in the main text. We consider the gauge condition with $\mathsf{G}_y = 1$. Then, Supplementary Eq.(7) becomes $\mathsf{G}_M(\boldsymbol{R})\mathsf{G}_M^*(L_y^{-1}(\boldsymbol{R})) = -1$, which means the period of gauge transformation is half of the lattice period along the y direction. We label the lattice sites by $(\alpha, \boldsymbol{R})$, where α labels sites within a unit cell and $\boldsymbol{R}$ coordinates unit cells. Because the gauge transformation $\boldsymbol{G}_M$ alternates its sign in the y direction, i.e., $\mathsf{G}_M(\alpha, \boldsymbol{R}) = -\mathsf{G}_M(\alpha, \boldsymbol{R} + \boldsymbol{e}_y)$, it transforms local states $|\alpha, \boldsymbol{R}\rangle$ as

$$|\alpha, \boldsymbol{R}\rangle \xrightarrow{\mathsf{G}_M} (-1)^{n_y}\mathsf{G}_{M\alpha}|\alpha, \boldsymbol{R}\rangle = e^{i\pi n_y}\mathsf{G}_{M\alpha}|\alpha, \boldsymbol{R}\rangle$$

$$= e^{i\boldsymbol{G}_y\cdot\boldsymbol{R}/2}\mathsf{G}_{M\alpha}|\alpha, \boldsymbol{R}\rangle, \tag{11}$$

where $\mathsf{G}_{M\alpha} = \mathsf{G}_M(\alpha, \mathbf{0})$ is the gauge transformation within a unit cell. The momentum-space states $|\alpha, \boldsymbol{k}\rangle$ are the Fourier transform of the real-space states, i.e., $|\alpha, \boldsymbol{k}\rangle = \sum_{\boldsymbol{R}} e^{i\boldsymbol{k}\cdot\boldsymbol{R}}|\alpha, \boldsymbol{R}\rangle$. Thus, G_M transforms the momentum-space states as

$$|\alpha, \boldsymbol{k}\rangle \xrightarrow{\mathsf{G}_M} \mathsf{G}_{M\alpha}|\alpha, \boldsymbol{k} + \boldsymbol{G}_y/2\rangle. \tag{12}$$

Hence, its action on the momentum space contains a half translation operator. Combining the reflection operator with the gauge transformation, the proper reflection in the momentum space is an glide-reflection,

$$\hat{\mathsf{M}}_x = U\mathcal{L}_{\frac{\boldsymbol{G}_y}{2}}\hat{m}_x. \tag{13}$$

For our model in the main context, in the absence of gauge fields, the momentum-space refection operator is $\hat{M}_x = \tau_0 \otimes \sigma_1 \hat{m}_x$. When we have gauge fields, only the signs of the intercell hopping amplitudes in the y direction are changed after reflection. Hence, the gauge transformation is simply given by $\mathsf{G}_M(\alpha, \boldsymbol{R}) = (-1)^{n_y}$. Accordingly, the momentum space symmetry operator is given by

$$\hat{\mathsf{M}}_x = \mathcal{L}_{\frac{\boldsymbol{G}_y}{2}}\hat{M}_x = \tau_0 \otimes \sigma_1\mathcal{L}_{\frac{\boldsymbol{G}_y}{2}}\hat{m}_x. \tag{14}$$

Supplementary Note 4. Screw Rotations in Momentum Space

In this section, we show how to realize screw rotations in momentum space. Without loss of generality, let us consider a rotation group C_n of order n in the xoy plane. In the absence of gauge field, the rotation generator R commutes with translation along z direction $RL_zR^{-1} = L_z$. When there are fluxes through the plaquettes spanned by L_z and R as illustrated in Supplementary Fig.1b, this equation is modified with an additional phase factor, i.e.,

$$\mathsf{R}\mathsf{L}_z\mathsf{R} = e^{i\Phi}\mathsf{L}_z. \tag{15}$$

The projective algebraic relation leads to

$$\begin{aligned}\mathsf{R}\mathsf{L}_z\mathsf{R}^{-1}\mathsf{L}_z^{-1} &= \mathsf{G}_R R\mathsf{G}_z L_z(\mathsf{G}_R R)^{-1}(\mathsf{G}_z L_z)^{-1}\\ &= \mathsf{G}_R(R\mathsf{G}_z R^{-1})RL_zR^{-1}L_z^{-1}(L_z\mathsf{G}_R L_z^{-1})\mathsf{G}_z^{-1}\\ &= \mathsf{G}_R(\boldsymbol{r})\mathsf{G}_z(R(\boldsymbol{r}))\mathsf{G}_R^*(L_z^{-1}(\boldsymbol{r}))\mathsf{G}_z^*(\boldsymbol{r}) = e^{i\Phi}.\end{aligned} \tag{16}$$

Consider the plaquette illustrated in Supplementary Fig.1b. According to Supplementary Eq. (4), the hopping amplitudes are related by

$$t_{24} = \mathsf{G}_R(2)\mathsf{G}_R^*(4)t_{13}, \quad t_{34} = \mathsf{G}_z(3)\mathsf{G}_z^*(4)t_{12}. \tag{17}$$

Then, we can substitute the identities into the formula of flux, which leads to some useful identities.

$$\begin{aligned}e^{-i\Phi} &= e^{i\phi_{12}}e^{i\phi_{24}}e^{i\phi_{43}}e^{i\phi_{31}}\\ &= \mathsf{G}_R(2)\mathsf{G}_R^*(4)\mathsf{G}_z^*(3)\mathsf{G}_z(4)e^{i\phi_{12}}e^{i\phi_{13}}e^{i\phi_{21}}e^{i\phi_{31}}\\ &= \mathsf{G}_R(2)\mathsf{G}_R^*(4)\mathsf{G}_z^*(3)\mathsf{G}_z(4)\\ &= \mathsf{G}_R(L_z^{-1}(4))\mathsf{G}_R^*(4)\mathsf{G}_z^*(R(4))\mathsf{G}_z(4).\end{aligned} \tag{18}$$

Because the rotation is n-fold, R^n is an identity operator, which means

$$\mathsf{L}_z = \mathsf{R}^n\mathsf{L}_z\mathsf{R}^{-n} = e^{in\Phi}\mathsf{L}_z. \tag{19}$$

Thus, Φ must be quantized, and therefore is valued in $\boldsymbol{Z}_n$, i.e., $\Phi = 2\pi m/n$ where m is an integer.

When $m \neq 0$, this projective algebraic relation can lead to a screw rotation in momentum space. In momentum space, we choose unit length c along z direction specified by L_z and gauge condition satisfies $\mathsf{G}_z = 1$, then the translation operator is diagonalized as $\hat{\mathsf{L}}_z = e^{ik_zc}$. The projective algebraic relation becomes

$$\hat{\mathsf{R}}e^{ik_zc}\hat{\mathsf{R}}^{-1} = e^{in\Phi}e^{ik_zc} = e^{i(k_zc+G_zm/n)}. \tag{20}$$

Thus, the proper transformation of R in momentum space contains a fractional translation on the reciprocal lattice, and R is represented as

$$\hat{\mathsf{R}} = U\mathcal{L}_{\frac{m\boldsymbol{G}_z}{n}}\hat{r}, \tag{21}$$

where $\mathcal{L}_{\frac{m\boldsymbol{G}_z}{n}}$ is the fractional translation on the reciprocal lattice, $\hat{r}$ rotates $\boldsymbol{k}$ vectors, and U is a unitary matrix.

With the screw-rotation in momentum space, the fundamental domain of Brillouin zone is $1/n$ of origin ones. But different from the glide-reflection, the screw rotations in momentum space preserve the orientation of the the Brillouin 3-torus, so the the topology of fundamental domain is still the same as 3-torus.

To illustrate the screw rotations in momentum space, we construct lattice models for $n = 2, 3, 4, 6$, which are, respectively, illustrated in Supplementary Fig.2abcd. It is noteworthy that these are all possible rotation degrees for crystalline systems. We choose an particular gauge condition: Only red bonds take a nontrivial hopping phase. When $n > 2$, let q be the number of arrow heads in each red bond. Then, the hopping amplitude is given by $t_{i,i+e_z} = e^{-iq\Phi}|t_{i,i+e_z}|$. Below, we list the model Hamiltonians and the corresponding symmetry operators in momentum space.

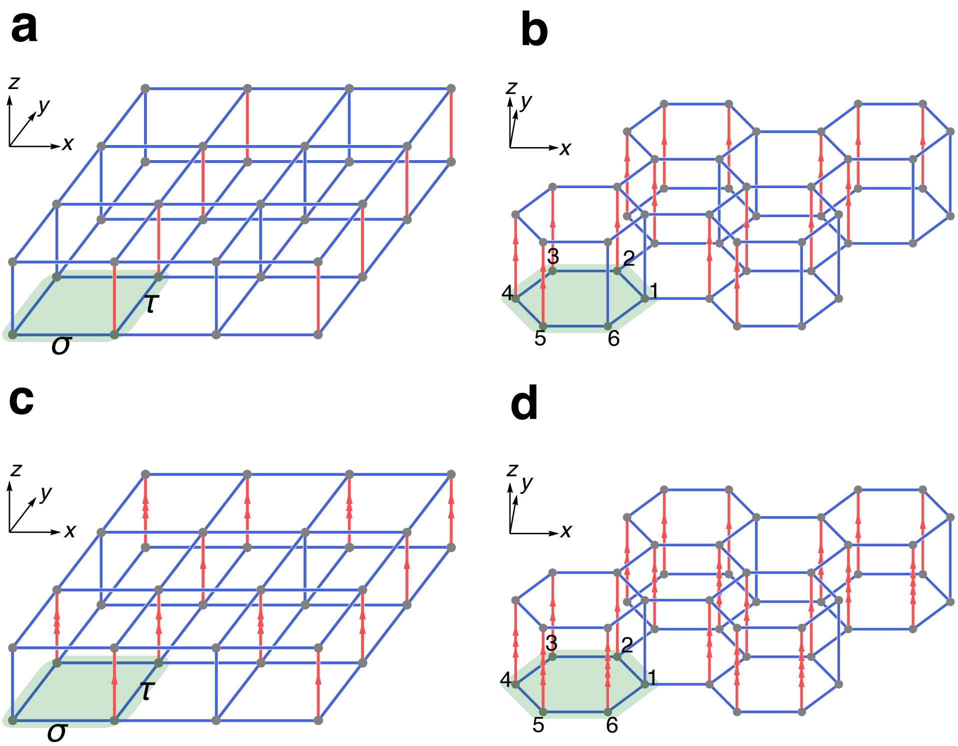

Supplementary Fig.2. **Lattice model with gauge connection satisfies n-folds screw rotation in the momentum space**. **a** $n = 2$. Flux in two nearby vertical surface surrounding the unit cell (green-shaded region) is π. **b** $n = 3$. Flux in two nearby vertical surface surrounding the unit cell is $\Phi = 2\pi m/3$ and $m = 1, 2$. **c** $n = 4$. Flux in each vertical surface surrounding the unit cell is $\Phi = \pi m/2$ and $m = 1, 2, 3$. **d** $n = 3$. Flux in each vertical surface surrounding the unit cell is $\Phi = \pi m/3$ and $m = 1, 2, 3, 4, 5$.

a $n = 2$. The rotation symmetry operator is represented by

$$\hat{\mathsf{R}}_\pi = \tau_1 \otimes \sigma_1 \mathcal{L}_{\frac{m\boldsymbol{G}_z}{2}}\hat{r}_\pi, \tag{22}$$

where $m = 1$, τ's and σ's are two sets of the Pauli matrices that operate on rows and columns of a unit cell. The Hamiltonian is given by

$$\mathcal{H}(\boldsymbol{k}) = \begin{bmatrix} 2t^z\cos k_z & t_1^x + t_2^x e^{-ik_x} & t_1^y + t_2^y e^{-ik_y} & 0 \\ t_1^x + t_2^x e^{ik_x} & 2t'^z\cos(k_z - \Phi) & 0 & t_1^y + t_2^y e^{-ik_y} \\ t_1^y + t_2^y e^{ik_y} & 0 & 2t'^z\cos k_z & t_1^x + t_2^x e^{-ik_x} \\ 0 & t_1^y + t_2^y e^{ik_y} & t_1^x + t_2^x e^{ik_x} & 2t^z\cos(k_z - \Phi) \end{bmatrix}. \tag{23}$$

b $n = 3$. The rotation symmetry operator is represented by

$$\hat{\mathsf{R}}_{2\pi/3} = U_{R_{2\pi/3}} \mathcal{L}_{\frac{m\boldsymbol{G}_z}{3}} \hat{r}_{2\pi/3}, \tag{24}$$

where $m = 1, 2$, and

$$U_{R_{2\pi/3}} = \begin{bmatrix} 0 & 0 & 1 & 0 & 0 & 0 \\ 0 & 0 & 0 & 1 & 0 & 0 \\ 0 & 0 & 0 & 0 & 1 & 0 \\ 0 & 0 & 0 & 0 & 0 & 1 \\ 1 & 0 & 0 & 0 & 0 & 0 \\ 0 & 1 & 0 & 0 & 0 & 0 \end{bmatrix}.$$

The Hamiltonian is given by

$$\mathcal{H}(\boldsymbol{k}) = \begin{bmatrix} 2t^z\cos(k_z) & t_1 & 0 & Je^{i\boldsymbol{k}\cdot\boldsymbol{a}_1} & 0 & t_2 \\ t_1 & 2t^z\cos(k_z-\Phi) & t_2 & 0 & Je^{i\boldsymbol{k}\cdot\boldsymbol{a}_2} & 0 \\ 0 & t_2 & 2t^z\cos(k_z-\Phi) & t_1 & 0 & Je^{i\boldsymbol{k}\cdot(-\boldsymbol{a}_1+\boldsymbol{a}_2)} \\ Je^{i\boldsymbol{k}\cdot\boldsymbol{a}_1} & 0 & t_1 & 2t^z\cos(k_z-2\Phi) & t_2 & 0 \\ 0 & Je^{i\boldsymbol{k}\cdot\boldsymbol{a}_2} & 0 & t_2 & 2t^z\cos(k_z-2\Phi) & t_1 \\ t_2 & 0 & Je^{i\boldsymbol{k}\cdot(-\boldsymbol{a}_1+\boldsymbol{a}_2)} & 0 & t_1 & 2t^z\cos(k_z) \end{bmatrix}, \tag{25}$$

where $\boldsymbol{a}_{1,2}$ are two lattice vectors in the xoy plane.

c $n = 4$. The The rotation symmetry operator is represented by

$$\hat{\mathsf{R}}_{\pi/2} = U_{R_{\pi/2}} \mathcal{L}_{\frac{m\boldsymbol{G}_z}{4}} \hat{r}_{\pi/2}, \tag{26}$$

where m= $1, 2, 3$ and

$$U_{R_{\pi/2}} = \begin{bmatrix} 0 & 1 & 0 & 0 \\ 0 & 0 & 0 & 1 \\ 1 & 0 & 0 & 0 \\ 0 & 0 & 1 & 0 \end{bmatrix}.$$

The Hamiltonian is given by

$$\mathcal{H}(\boldsymbol{k}) = \begin{bmatrix} 2t^z\cos k_z & t_1+t_2e^{-ik_x} & t_1+t_2e^{-ik_y} & 0 \\ t_1+t_2e^{ik_x} & 2t^z\cos(k_z-\Phi) & 0 & t_1+t_2e^{-ik_y} \\ t_1+t_2e^{ik_y} & 0 & 2t^z\cos(k_z-3\Phi) & t_1+t_2e^{-ik_x} \\ 0 & t_1+t_2e^{ik_y} & t_1+t_2e^{ik_x} & 2t^z\cos(k_z-2\Phi) \end{bmatrix}. \tag{27}$$

d $n = 6$. The rotation symmetry operator is represented by

$$\hat{\mathsf{R}}_{\pi/3} = U_{R_{\pi/3}} \mathcal{L}_{\frac{m\boldsymbol{G}_z}{6}} \hat{r}_{\pi/3}, \tag{28}$$

where $m = 1, 2, 3, 4, 5$ and

$$U_{R_{\pi/3}} = \begin{bmatrix} 0 & 1 & 0 & 0 & 0 & 0 \\ 0 & 0 & 1 & 0 & 0 & 0 \\ 0 & 0 & 0 & 1 & 0 & 0 \\ 0 & 0 & 0 & 0 & 1 & 0 \\ 0 & 0 & 0 & 0 & 0 & 1 \\ 1 & 0 & 0 & 0 & 0 & 0 \end{bmatrix}.$$

The Hamiltonian is given by

$$\mathcal{H}(\boldsymbol{k}) = \begin{bmatrix} 2t^z\cos(k_z) & t & 0 & Je^{i\boldsymbol{k}\cdot\boldsymbol{a}_1} & 0 & t \\ t & 2t^z\cos(k_z-\Phi) & t & 0 & Je^{i\boldsymbol{k}\cdot\boldsymbol{a}_2} & 0 \\ 0 & t & 2t^z\cos(k_z-2\Phi) & t & 0 & Je^{i\boldsymbol{k}\cdot(-\boldsymbol{a}_1+\boldsymbol{a}_2)} \\ Je^{i\boldsymbol{k}\cdot\boldsymbol{a}_1} & 0 & t & 2t^z\cos(k_z-3\Phi) & t & 0 \\ 0 & Je^{i\boldsymbol{k}\cdot\boldsymbol{a}_2} & 0 & t & 2t^z\cos(k_z-4\Phi) & t \\ t & 0 & Je^{i\boldsymbol{k}\cdot(-\boldsymbol{a}_1+\boldsymbol{a}_2)} & 0 & t & 2t^z\cos(k_z-5\Phi) \end{bmatrix}. \tag{29}$$

Supplementary Note 5. Numerical Demonstration of Stability

To demonstrate the stability of the topology with the addtion of atomic energy bands, we add an orbit in the middle of the unit cell as fig.3.a. The addition preserves the symmetry of our model since we put it on the wyckoff position of reflection. The atomic orbit has on site energy ε' and couplings δ to other nearest orbits. The Hamiltonian now becomes

$$\bar{\mathcal{H}}(\boldsymbol{k}) = \begin{bmatrix} \varepsilon & [q_1^x(k_x)]^* & [q_+^y(k_y)]^* & 0 & \delta \\ q_1^x(k_x) & \varepsilon & 0 & [q_-^y(k_y)]^* & \delta \\ q_+^y(k_y) & 0 & -\varepsilon & [q_2^x(k_x)]^* & \delta \\ 0 & q_-^y(k_y) & q_2^x(k_x) & -\varepsilon & \delta \\ \delta & \delta & \delta & \delta & \varepsilon' \end{bmatrix}. \tag{30}$$

If we take $\varepsilon' = -2, \delta = 0$, and other parameters same with fig.4d in the main context, the atomic energy band merge into the valence band as Supplementary Fig.3.b. If we turn on the coupling strength δ, we can see that the topological edge bands remain stable as fig3.c.

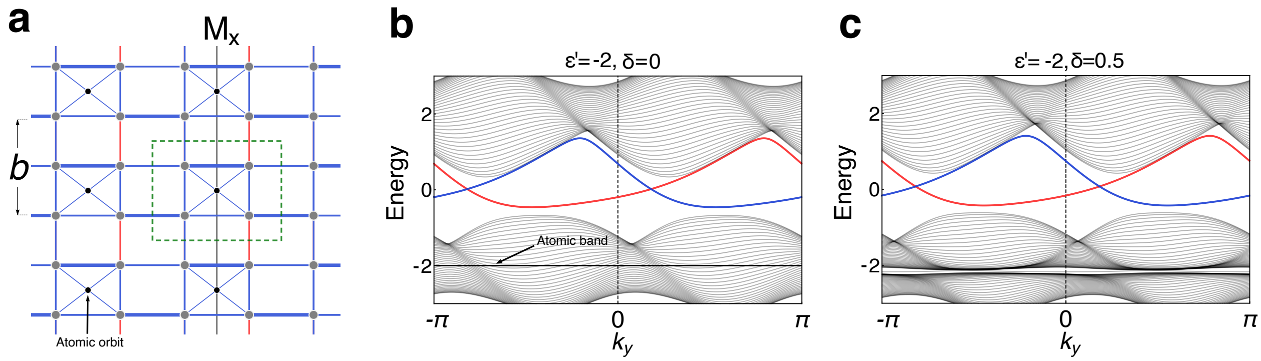

Supplementary Fig.3. **Stability of topology with adding of trivial bands**. **a** The addition of an atomic orbit in the middle of unit cell. **b** and **c** depict the corresponding band structures on a ribbon geometry with edges along y. We take the on site energy and coupling of the atomic band as $\varepsilon' = -2, \delta = 0$ and $\varepsilon' = -2, \delta = 0.5$ in **b** and **c** respectively.

Supplementary Note 6. Gauge Fields in Physical Systems

In this section, we present a brief review on gauge fields in physical systems.

For condensed matter system, $\boldsymbol{Z}_2$ gauge fields can appear in quantum spin liquids. In the mean-field theory of quantum spin liquids, close to the ground states the spinors are coupled to gauge fields, for instance a $\boldsymbol{Z}_2$ gauge field (which defines $\boldsymbol{Z}_2$ spin liquids). In fact, probably this is the first time physicists noticed given gauge flux configurations can lead to projective representations of space groups (PSG), and thereby Xiao-Gang Wen proposed the PSG classification scheme for quantum phases of spin liquids.

We would like to point out a significant fact, i.e., $\boldsymbol{Z}_2$ gauge fields are essentially different from U(1) gauge fields in that time-reversal symmetry is preserved by $\boldsymbol{Z}_2$ gauge fields. This can be directly seen from the fact that real hopping amplitudes are sufficient to implement all $\boldsymbol{Z}_2$ gauge flux configurations, which naturally preserves time-reversal symmetry. More fundamentally, time reversal inverses flux, but $\pi = -\pi$ on a lattice. Thus, it is possible to realize $\boldsymbol{Z}_2$ gauge fields without introducing magnetic fields. While there could be other mechanisms, here we note the so-called dark-bright mechanism. The low-energy effective hopping amplitude of two sites through an intermediate high-energy site is negative $-t^2/\Delta$, with Δ the energy gap. This mechanism suggests that certain second-order hopping amplitudes can also take a negative phase in non-interacting crystalline systems.

In artificial systems, gauge fields can be engineered. This is an advantage for realizing our theoretical proposal. Below, we briefly introduce the mechanisms for generating gauge fields in these artificial systems.

- In photonic crystals, the artificial gauge field can be directly simulated by modulation of the resonant frequencies, i.e., adjusting the gap between site ring and link-ring wave guides. Other method is shaking the photonic crystal and the effective gauge field results from Floquet's theorem, similar to the cold atoms.

- In acoustic crystals, $\boldsymbol{Z}_2$ hopping phases can be readily realized by coupling the resonators with wave guides on different sides.
- In cold atom systems, the approaches of simulating the gauge field include rotating optical lattice, laser-assisted tunneling, and shaking the optical lattice. (i) Rotating optical lattice can only introduce the weak and uniform effective magnetic field and the side effect of Coriolis force should be compensated. (ii) For the laser-assisted tunneling, the atomic hopping with desired gauge potentials are engineered by coupling internal levels of atoms with laser beams and thus different kinds of gauge fields can be simulated even including the nonabelian one. (iii) The advantage of shaking optical lattice relies on that it is free of internal levels.
- In mechanical systems, effective $\boldsymbol{Z}_2$ gauge field can be simulated by just tuning the stiffness coefficients of the springs.
- For the electric circuits, the $\boldsymbol{Z}_2$ gauge field can be realized by tuning the capacitances and inductances.
